# Complete pathological response following neoadjuvant FOLFIRINOX in borderline resectable pancreatic cancer - a case report and review

**DOI:** 10.1186/s12885-016-2821-0

**Published:** 2016-10-10

**Authors:** Mišo Gostimir, Sean Bennett, Terence Moyana, Harman Sekhon, Guillaume Martel

**Affiliations:** 1 Faculty of Medicine, University of Ottawa, 451 Smyth Rd, K1H 8 M5 Ottawa, Canada; 2 Department of Surgery, Division of General Surgery, University of Ottawa, 451 Smyth Rd, K1H 8 M5 Ottawa, Canada; 3 Department of Pathology and Laboratory Medicine, University of Ottawa, 501 Smyth Rd, K1H 8 L6 Ottawa, Canada; 4 Department of Surgery, Liver and Pancreas Unit, University of Ottawa, 501 Smyth Rd, K1H 8 L6 Ottawa, Canada

**Keywords:** FOLFIRINOX, Pancreatic cancer, Complete pathological response, Neoadjuvant therapy, Locally-advanced, Borderline resectable, Case report

## Abstract

**Background:**

Pancreatic cancer is among the top 5 most common cancers worldwide, but is particularly devastating due to its insidious nature. Complete surgical resection remains the only potential curative treatment, although only 20 % of patients present with a resectable tumor. Patients may alternatively present with borderline resectable pancreatic cancer or locally advanced pancreatic cancer and can be offered treatment with neoadjuvant intent. The effectiveness of these treatments is unclear and there is a paucity of data to suggest one optimal treatment approach.

**Case presentation:**

We describe a 61-year-old female who presented with a two-week history of obstructive jaundice in the context of vague abdominal pain that had been ongoing for years prior to her visit. CT scan of the abdomen confirmed a hypovascular mass in the uncinate process consistent with borderline resectable pancreatic cancer. Pancreatic adenocarcinoma was confirmed with endoscopic ultrasound guided fine-needle aspiration cytology. Following multidisciplinary discussion, it was recommended that she undergo treatment with FOLFIRINOX. After a total of 13 cycles, follow up CT revealed that the lesion had decreased in size and she was offered resection as a potentially curative treatment. She underwent pancreaticoduodenectomy. Final pathology report revealed no evidence of residual adenocarcinoma (ypT0 ypN0 (0/23)). The patient remains disease-free 15 months following surgery.

**Conclusion:**

To date, there have been very few reports of a complete pathological response following neoadjuvant therapy in borderline resectable or locally advanced pancreatic cancer. This report describes a unique case of a complete pathological remission in a patient with borderline resectable pancreatic cancer following FOLFIRINOX therapy alone and adds to the growing base of evidence meriting the initiation of clinical trials to assess the efficacy of FOLFIRINOX in these subsets of pancreatic cancer.

## Background

Pancreatic adenocarcinoma is one of the top five causes of cancer death worldwide. It is particularly devastating due to its insidious progression. The red flag symptoms prompting diagnosis often appear only once the disease has already progressed or metastasized. As a result, only 10–20 % of pancreatic cancers are resectable at the time of presentation and the overall 5-year survival rate is less than 5 % [1, 2].

Early intervention in non-metastatic pancreatic cancer is crucial as metastases have been shown to occur unexpectedly before clinical detection is possible [3–5]. Surgical resection is thought to be the only curative treatment for pancreatic adenocarcinoma and offers a five year survival rate of 10–15 % [1, 6, 7]. Neoadjuvant chemotherapy is often implemented in an effort to increase the incidence of R0 resections, reduce local recurrences, treat occult micrometastases, and to downstage a borderline resectable tumor to the point of possible resection [1].

A subset of patients is considered to have borderline resectable pancreatic cancer, which can be classified using three main staging systems. The first staging systems were established by M.D. Anderson and the National Comprehensive Cancer Network. A third system was put forth by the Americas Hepatopancreatobiliary Association/Society of Surgical Oncology/Society for Surgery of the Alimentary Tract in 2009 and was ultimately adopted by the National Comprehensive Cancer Network. Based on the M.D. Anderson criteria, borderline resectability can be defined as tumor abutment of ≤ 180° of the circumference of the superior mesenteric artery (SMA), short-segment encasement or abutment of the common hepatic artery (typically at the gastroduodenal origin), or short-segment occlusion of the superior mesenteric vein or portal vein with suitable vessels above and below [8]. The current National Comprehensive Cancer Network system is largely similar to the M.D. Anderson criteria except that abutment (≤180°) or encasement (>180°) of the superior mesenteric vein/portal vein without vein contour irregularity is considered borderline resectable. Thus, non-occlusive involvement of the superior mesenteric vein or portal vein is considered only borderline resectable by the National Comprehensive Cancer Network criteria, but resectable by the M.D. Anderson criteria [8–11]. Patients with borderline resectable pancreatic cancer typically undergo chemotherapy and/or chemoradiation as a neoadjuvant approach, followed by radical surgical resection.

About 30 % of patients have locally advanced pancreatic cancer [2, 5] which is defined by the M.D. Anderson criteria as tumor encasement of the SMA beyond 180°, encasement of the celiac artery or hepatic artery, or occlusion of the superior mesenteric vein or portal vein, all in the absence of metastatic disease [8]. Patients with locally advanced pancreatic cancer are generally treated with chemotherapy or chemoradiation. In the majority of patients, this is considered palliative treatment, although a small subset of patients who see a radiological response may eventually be considered for surgical resection.

Pathologic complete response (pCR) is used to refer to a neoplastic tissue specimen with no residual viable invasive adenocarcinoma cells within the parenchyma [12]. For several different cancer types, pCR is associated with lower frequencies of local recurrences, distant metastases, and overall better survival rates. The significance of pCR in pancreatic cancer has also been demonstrated by an association with high survival rates [12, 13]. To our knowledge, while there have been several reports of a pCR following neoadjuvant therapy in borderline resectable or locally advanced pancreatic cancer [5, 14–20], there has been only one case report of a patient who achieved pCR with FOLFIRINOX alone [21]. This article describes the second report of a well documented, histologically proven pCR following systemic treatment with FOLFIRINOX.

## Case presentation

A 61-year-old French Canadian female presented to hospital with a two-week history of obstructive jaundice, pruritus, tea-coloured urine, acholic stools, fatigue, anorexia, and unintentional weight loss. Her history also included vague abdominal pain that had been ongoing for years prior to her visit, although the pattern of pain did not change with the onset of jaundice. Her past medical history included type 2 diabetes mellitus and hypertension. She did not have a personal history of cancer, but her family history included two aunts who had pancreatic cancer in their sixth decades of life. She denied any tobacco, alcohol, or recreational drug use.

**Fig. 1:**
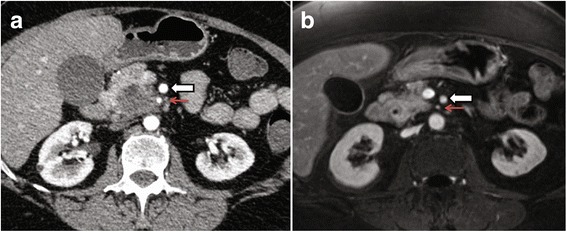
Abdominal CT scans. **a** The mass is separate from the celiac axis an there is loss of a fat plane between the mass and SMA (*white arrow)* with less than 90° involvement of its circumference. The first arterial jejunal branch (*red arrow*) is also encased with tumor. There is also mild narrowing of the medial aspect of the superior mesenteric vein where the tumor abuts around 90° of its circumference. The pancreatic duct is dilated, measuring up to 6 mm. **b** Following 9 cycles of FOLFIRINOX, the follow up MRI reveals that the head of the pancreas has indistinct margins, but has clearly diminished in size. The measured size is 2.7 × 2.3 mm (prior: 3.9 × 3.2 mm). Pancreatic ductal dilatation has subsided, indicating response to treatment

Abdominal ultrasound revealed a lesion in the uncinate process of the pancreas. A subsequent CT scan of the abdomen confirmed a fairly well circumscribed hypovascular mass in the head and uncinate process of the pancreas, radiographically consistent with pancreatic adenocarcinoma (Fig. 1a). The mass encircled the first jejunal branch of the superior mesenteric artery by approximately 180°. Endoscopic retrograde cholangiopancreatography (ERCP) was ultimately unsuccessful due to inability to cannulate the common bile duct. The pancreatic duct was patent and could be cannulated but seemed to end abruptly.

**Fig. 2:**
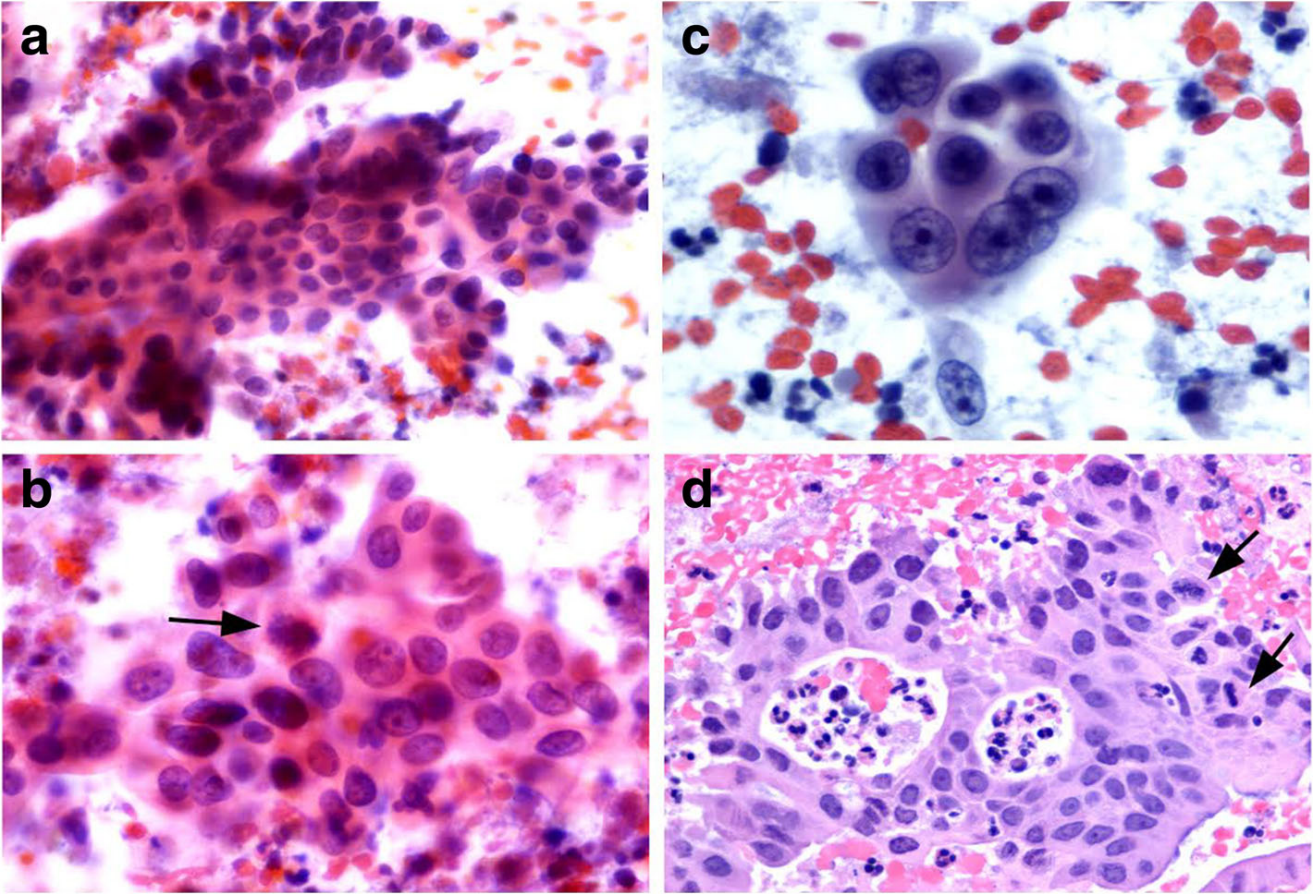
Photomicrographs showing cytomorphological findings of endoscopic ultrasound guided fine needle aspirate of pancreatic adenocarcinoma. **a** Cellular aspirate depicting glandular cells arranged in complex architecture (Papanicolaou stain, magnification 200×). **b** and **c** The cells are markedly pleomorphic with enlarged irregular nuclei, macronucleoli and moderate lacy cytoplasm (magnification 400× and 600×). **d** Section of cell block show markedly atypical cells disposed in cribriform architecture. Frequent mitotic figures (*black arrows*) are also present (magnification 400×)

The patient underwent an endoscopic ultrasound, which revealed a 4.8 × 3.5 cm hypoechoic mass in the pancreatic head/uncinate process along with numerous cystic changes in the body, tail, and distal tail of the pancreas, measuring 2.4 mm, 3.9 mm, and 4.3 mm, respectively. Fine-needle aspiration (FNA) biopsy at the time of endoscopic ultrasound demonstrated cytology consistent with adenocarcinoma (Fig. 2).

At the time of diagnosis, the patient’s functionality was consistent with Eastern Cooperative Oncology Group (ECOG) grade 1: restricted in physically strenuous activity, but ambulatory and able to carry out light work. The CA19-9 levels were ordered but deferred by the patient. Her total bilirubin at the time of diagnosis was 160 μmol/L. The patient underwent placement of a percutaneous transhepatic biliary drainage catheter, with resolution of her jaundice. The patient was reviewed at a weekly hepatobiliary and pancreatic cancer multidisciplinary conference. Consensus was that her tumor was borderline resectable on the basis of partial encasement of the superior mesenteric artery. Although there was encasement of the first jejunal branch, the tumor was staged based on abutment of less than ≤ 180° of the SMA. Radiologically, the patient was staged as T3 N0 M0. It was recommended that the patient begin systemic chemotherapy with four cycles of FOLFIRINOX (Irinotecan 180 mg/m^2^; Oxaliplatin 85 mg/m^2^; 5-Fluorouracil 400 mg/m^2^; Folinic acid 400 mg/m^2^), after which she would be re-imaged for assessment of treatment efficacy.

The patient tolerated the four cycles without undue toxicity, after which she underwent re-staging with CT scans of the chest, abdomen, and pelvis. This revealed multiple non-calcified small pulmonary nodules detected bilaterally in the lower lobes, the largest of which measured 4 mm. These nodules were previously not present and were thus considered suspicious for metastatic disease. As a result of the equivocal pulmonary nodules, resection was not indicated and the decision was made to continue FOLFIRINOX to further assess both local response, and the response of the potential metastatic nodules.

The patient received a further nine cycles of FOLFIRINOX. Follow up MRI revealed that the pancreatic lesion had impressively decreased in size from 3.9 × 3.2 cm to 2.7 × 2.3 cm compared to the previous CT (Fig. 1b). Furthermore, the lung lesions had remained stable since the last CT scan, raising the possibility that they were not metastatic. Given these results, and after discussion at multidisciplinary cancer conference, a resection was offered to the patient as a potentially curative treatment. She continued two more cycles of therapy before undergoing a pancreaticoduodenectomy, six weeks following discontinuation of FOLFIRINOX and 33 weeks from the date of diagnosis. No major technical difficulties were encountered in the procedure, although there was considerable tissue edema as expected from the chemotherapy. No vascular resection or reconstruction was required. Her postoperative course was complicated by pneumonia. She was discharged home on day 11. She was readmitted 9 days later with acute kidney injury, secondary to vomiting, poor intake and diarrhea, requiring one round of hemodialysis (peak creatinine 847 μmol/L or 9.58 mg/dL, Dindo-Clavien grade 4a).

**Fig. 3:**
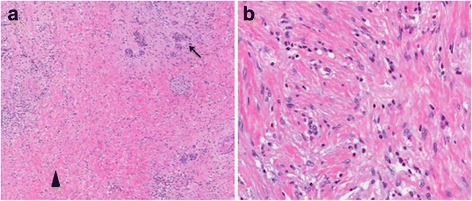
Photomicrographs showing the cytomorphological findings of pancreatic specimens following surgical resection. **a** Low power view of section from resected pancreas showing residual acini and islets (*arrow*) while the previous tumor bed (*arrowhead*) shows fibrosis and a repair reaction. (Hematoxylin eosin; original magnification × 40). **b** Higher magnification from the fibrotic area showing fibroblasts laying down collagen and a scattering of inflammatory cells (Hematoxylin eosin; original magnification × 200)

Histological examination of the surgical specimen revealed no evidence of residual adenocarcinoma (Fig. 3), consistent with a complete response to treatment (College of American Pathologists grade 0) [22]. As is required in this situation, the entire specimen was submitted for histological assessment, confirming the complete pathological response (ypT0). Severe acute and chronic pancreatitis and areas of fibrosis were noted, together with scattered foci of grade 1 pancreatic intraepithelial neoplasia. All 23 resected lymph nodes were negative for malignancy (ypN0).

Following surgery, adjuvant therapy was not given. Her CT scans at 3 and 6 months postoperatively show no evidence of recurrence, with no appreciable change in her previously noted lung nodules. Her CA 19–9 is normal at 20 kU/L. She remains disease-free 15 months after surgery, and 24 months after diagnosis. Given her strong family history of pancreatic cancer, she was referred for genetic testing. She was found to be a carrier of a BRCA2 mutation that is not uncommon among French Canadians (BRCA2: E3002K).

## Discussion

The introduction of chemotherapy and radiotherapy as a possible neoadjuvant option in borderline resectable and locally advanced pancreatic cancer has yielded some positive results for patients with tumors that would have otherwise been unresectable. In a review of 111 studies, Gillen et al. concluded that approximately one-third of patients with initially unresectable locally advanced pancreatic cancer could become resectable following neoadjuvant therapy consisting of radiotherapy, chemotherapy, or a combination of the two [23]. Furthermore, successful resection following neoadjuvant therapy in locally advanced pancreatic cancer offers a median survival of 20.5 months, similar to that of R0 resections in patients with initially resectable cases of pancreatic cancer [23]. Despite these positive results, a margin-positive resection conversely offers survival rates similar to that of patients with locally advanced pancreatic cancer who were treated with palliative intent as well as those who had metastatic disease at presentation [23]. Thus, the optimal management approach in locally advanced pancreatic cancer remains unclear, as there is currently only limited evidence to support neoadjuvant approaches.

In taking a neoadjuvant approach for locally advanced pancreatic cancer, the goal of treatment is to increase the likelihood of R0 resection, to obliterate micrometastatic disease, and to select for patients with favorable tumor biology by excluding those who progress on therapy [8]. Commonly used regimens have included gemcitabine alone, combination gemcitabine-platinum, as well as chemoradiotherapy using 5-FU or gemcitabine as chemosensitizers [24]. The optimal neoadjuvant approach in borderline resectable pancreatic cancer is still under investigation, but similarly to locally advanced pancreatic cancer, common approaches involve chemoradiotherapy or initial chemotherapy followed by radiotherapy [25]. The latter approach minimizes toxic side effects of radiotherapy and selects for patients without early metastasis while also offering potential tumour reduction [26]. At our institution, patients with both borderline resectable and locally advanced pancreatic cancer are managed initially with FOLFIRINOX as a potentially neoadjuvant approach. Patients are re-imaged after 4–6 cycles. To be considered an operative candidate, patients with borderline resectable pancreatic cancer must demonstrate stable disease or regression, while patients with locally advanced pancreatic cancer must demonstrate regression. These decisions are all made at a multidisciplinary cancer conference. Most of the existing trials for neoadjuvant therapy have included chemoradiotherapy and at this point the relative contributions of chemotherapy and radiotherapy are not well understood [27–33].

FOLFIRINOX represents a new generation of efficacious combination chemotherapy regimens that may be utilized to treat patients with similar cases of pancreatic cancer. The efficacy of FOLFIRINOX was first demonstrated in a study by Conroy et al. in patients with unresectable pancreatic cancer [34]. A subsequent trial reported a median survival of 11.1 months in patients with metastatic pancreatic cancer who had received FOLFIRINOX as compared to a median survival of 6.8 months in patients receiving gemcitabine [35]. However, despite these results, the uptake of FOLFIRINOX into the arsenal of therapeutic options for pancreatic cancer has been slow due to concerns of toxicity. This is especially the case for locally advanced pancreatic cancer due to the paucity of data supporting FOLFIRINOX as a neoadjuvant option for this type of potentially resectable pancreatic cancer.

There is much hope that FOLFIRINOX could usher in a new era of downstaging of borderline resectable pancreatic cancer, allowing R0 resection. In a study of 18 patients with borderline resectable pancreatic cancer who were given FOLFIRINOX, 12 patients underwent pancreatectomy with negative margins [36]. However, this report is the first to describe a patient who achieved pCR with FOLFIRINOX alone. One example, reported in 2013 by Hartlapp et al., described a patient with locally advanced pancreatic cancer treated initially with nab-paclitaxel and gemcitabine which was eventually switched to FOLFIRINOX alone, resulting in pCR [5]. In their report, nab-paclitaxel and gemcitabine ultimately led to an increase in the level of CA19-9, prompting their switch to FOLFIRINOX. Pathological analysis of the pancreatic head in their patient revealed no viable cancer cells but the specimen did contain residual pancreatic intraepithelial neoplasia. Of note, Hartlapp et al. speculated that their preceding use of nab-paclitaxel might have played a vital role in increasing delivery of the subsequent FOLFIRINOX into the tumor cells.

In 2015, Valeri et al. were the first to report a patient with locally advanced pancreatic cancer who achieved pCR following neoadjuvant treatment with FOLFIRINOX alone (Table 1) [21]. Their patient had a locally advanced cancer of the pancreatic head that was treated with 8 cycles of FOLFIRINOX until restaging by CT scan demonstrated complete disappearance of the tumor [21]. Of note, the preoperative histology results for their patient described an undifferentiated carcinoma, which is a rare malignant epithelial neoplasm with dismal survival rates [21]. In a 2013 study of 25 patients with either unresectable or borderline resectable disease, Boone et al. also achieved pCR in one patient, although the details of this patient were not reported [14]. In a 2015 study by Blazer et al. of 25 locally advanced and 18 borderline resectable pancreatic cancer patients receiving modified FOLFIRINOX regimens, radiographic complete response was obtained in 9 patients. However, this study also included some patients who received radiation therapy. The outcomes were not sub-divided by treatment group, and therefore the effects of FOLFIRINOX alone could not be extrapolated [37].

**Table 1:** Outcomes of neoadjuvant FOLFIRINOX regimens in locally advanced and borderline resectable pancreatic cancer

Authors	Journal, Year	Number of Patients	Staging System	Duration (Cycles)	Radiographic Response	Surgical Resection	R0 Rate	Pathological Results
Blazer et al.	Ann Surg Oncol 2015	25 LAPC 18 BRPC	AHPBA/ SSO/ SSAT	4.9 (mean)	NA	NA	NA	NA
Boone et al. [14]	Surg Oncol, 2013	13 LAPC 12 BRPC	AHPBA/ SSO/ SSAT	5 (mean)	PD 1 Intolerable SE 1	5/11	4/5	1 CAP g0 4 CAP g1
Gunturu et al. [54]	Med Oncol, 2013	16 LAPC	NR	11 (median)	PR 8 SD 7 PD 0	2/16	NR	1 near pCR (2 mm residual tumor)
Hosein et al. [55]	BMC Cancer, 2012	14 LAPC 4 BRPC	AHPBA/ SSO/ SSAT	6 (median)	LAPC (1 PD, 9 RT) BRPC (1 RT)	LAPC (3/14) BRPC (3/4)	LAPC (2/3) BRPC (3/3)	NR
Khushman et al. [56]	Pancreatology, 2015	25 LAPC	AHPBA/ SSO/ SSAT	8 (median)	PD 2	10/25	7/10	NR
Nitsche et al. [18]	Ann Surg Oncol, 2015	14 LAPC/ BRPC	NCCN	7 (median)	6 PR, 6 SD, 1 PD	0/14	NR	NR
Peddi et al. [57]	JOP, 2012	19 LAPC 4 BRPC	NR	4 (median)	1 rCR, 5 PR, 9 SD, 3 PD	4/23	NR	NR
Valeri et al. [21]	Pancreatology, 2014	1 LAPC	MDA	8	NR	1/1	1/1	1 pCR

*LAPC* locally advanced pancreatic cancer*, BRPC* borderline resectable pancreatic cancer, *AHPBA* Americas Hepatopancreatobiliary Association, *SSO* Society of Surgical Oncology, *SSAT* Society for Surgery of the Alimentary Tract, *NA* not available, *PD* progressive disease*, SE* side effects, *PR* partial remission, *SD* stable disease, *rCR* radiological complete response, *CAP* College of American Pathologists grading system, *NR* not reported, *NCCN* National Comprehensive Cancer Network*, MDA* M.D. Anderson

Complete pathological response in the context of pancreatic cancer is a rare outcome. The probability of pCR following various neoadjuvant therapy approaches in pancreatic cancer has been shown to be 3.6 %, while the partial response rate is 30.6 % [23]. In patients with borderline resectable or locally advanced pancreatic cancer, neoadjuvant therapy has been shown to lead to pCR in 13.6 % of patients [38]. However, these studies considered all forms of neoadjuvant therapy and currently, most existing reports of pCR in pancreatic cancer involve chemoradiation rather than chemotherapy alone [6, 15–20, 39–49]. Other reports of complete responses have been only radiologically confirmed [34, 50–52]. There are presently multiple definitions for locally advanced and borderline resectable pancreatic cancer which have made it difficult to extrapolate conclusions on the efficacy of FOLFIRINOX as most current studies include heterogeneous patient populations [53].

## Conclusion

The current work presented a rare occurrence of complete pathological response in a patient with borderline resectable pancreatic cancer following treatment with FOLFIRINOX. Consistent adoption and reporting of resectability classification in the initial staging of pancreatic cancers will allow for more homogenous study populations and a better assessment of the impact of neoadjuvant therapies in borderline resectable and locally advanced pancreatic cancer.

## Abbreviations

SMA: Superior mesenteric artery
PCR: Complete pathological response
ERCP: Endoscopic retrograde cholangiopancreatography
FNA: Fine-needle aspiration
CAP: College of American Pathologists grading system
